# Depressive Symptoms Are the Dominant Independent Correlate of Patient-Reported Cognitive Function in Older Women with Locally Advanced Breast Cancer: A Cross-Sectional Geriatric Assessment Study

**DOI:** 10.3390/jcm15145655

**Published:** 2026-07-19

**Authors:** Merve Tokocin, Kayra Cangoz, Huseyin Kadioglu

**Affiliations:** 1 Department of General Surgery, Istanbul Bagcilar Training and Research Hospital, 34200 Istanbul, Turkey; 2 Department of General Surgery, Akdagmadeni Sehit Sinan Babacan State Hospital, 66300 Yozgat, Turkey; 3 Department of General Surgery, Kanuni Sultan Suleyman Training and Research Hospital, 34303 Istanbul, Turkey; huseyinkadioglu@gmail.com

**Keywords:** geriatric oncology, breast cancer, frailty, depression, cognitive function

## Abstract

**Background/Objectives**: Older women with breast cancer carry a high burden of frailty, depressive symptoms and cognitive complaints, yet how these domains relate to one another in routine clinical practice remains incompletely understood. We examined the prevalence of positive frailty screening and its association with patient-reported cognitive function and asked whether depressive symptoms account for the apparent link between the two. **Methods**: We analyzed 314 women aged 65 years or older with stage IIB–IIIC breast cancer who underwent a standardized geriatric assessment. Measures included the G8 screening tool, Vulnerable Elders Survey-13 (VES-13), Geriatric Depression Scale (GDS), Instrumental Activities of Daily Living (IADL), cognition and quality of life (FACT-Cog, FACT-G, and EORTC QLQ-C30). Associations were examined using correlation analyses, group comparisons, and multivariable linear regression. **Results**: Mean age was 72.9 years. A positive frailty screen (G8 ≤ 14) was present in 295 patients (93.9%), and 102 patients (32.5%) screened positive for depressive symptoms (GDS ≥ 5). G8 screening score correlated strongly with patient-reported cognitive function (Pearson r = 0.80, *p* < 0.001), and patients with a positive frailty screen reported lower FACT-Cog scores than those with a negative screen (113.0 vs. 129.0, *p* < 0.001). In multivariable analysis adjusted for age and G8 score, depressive symptoms emerged as the strongest independent correlate of FACT-Cog (β = −0.89, *p* < 0.001; model R^2^ = 0.90), whereas G8 screening score was no longer independently associated (*p* = 0.16). Patients with depressive symptoms had more than double the VES-13 vulnerability score of those without (7.8 vs. 3.6, *p* < 0.001). Overall geriatric assessment profiles were generally comparable across the three exploratory treatment-era cohorts. **Conclusions**: In this cross-sectional analysis of older women with locally advanced breast cancer, depressive symptoms were the strongest independent correlate of patient-reported cognitive function. Incorporating a brief depression screen into routine geriatric assessment may represent a clinically actionable target that deserves further evaluation.

## 1. Introduction

Breast cancer predominantly affects older women, with an increasing proportion of patients aged 70 years or older, many of whom present with locally advanced disease requiring multimodal treatment. Chronological age alone is a poor guide to how these patients tolerate therapy, and for this reason, major oncology societies now recommend that older patients undergo some form of geriatric assessment before treatment decisions are made [1,2,3]. This is particularly relevant given that older patients with cancer frequently prioritize quality of life and preserved independence over survival alone and express a strong wish to avoid becoming a burden to family or carers [4].

Three domains in particular have been shown to shape clinical outcomes in this population: physical frailty, depressive symptoms and cognitive difficulties. Each has been studied individually, and each predicts poorer treatment tolerance, greater functional decline and worse quality of life [5,6,7]. What is less clear is how they interact in routine practice.

**Figure A1:**
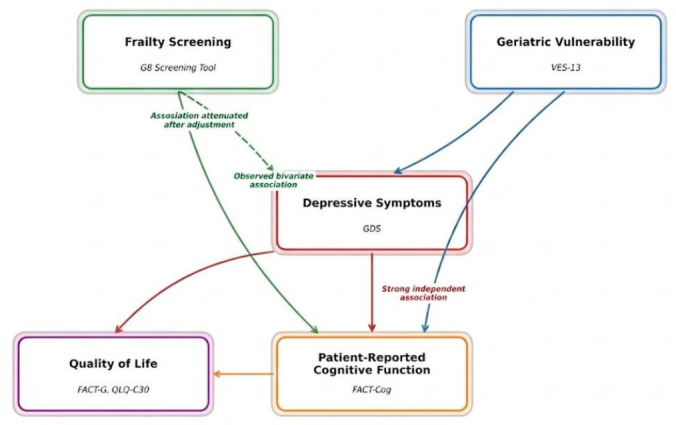
Conceptual Framework. Conceptual framework illustrating the hypothesized relationships among frailty screening, geriatric vulnerability, depressive symptoms, patient-reported cognitive function, and quality of life. Solid arrows represent relationships evaluated in the present study, whereas the dashed arrow denotes the hypothesized direct association between frailty screening and patient-reported cognitive function after adjustment for depressive symptoms. The framework was developed a priori to guide the study hypothesis and statistical analyses and should not be interpreted as implying causality.

Frailty and vulnerability are often used interchangeably in clinical oncology despite representing related but distinct constructs. Frailty reflects diminished physiological reserve and increased vulnerability to adverse health outcomes, extending beyond chronological age alone. In older adults with cancer, it is associated with poorer treatment tolerance, postoperative complications, functional decline, and reduced survival. Accordingly, current geriatric oncology guidelines recommend frailty screening as an initial step, followed by a comprehensive geriatric assessment when indicated to guide individualized treatment decisions. In this study, frailty was screened using the G8 questionnaire, a validated tool that identifies patients who may benefit from comprehensive geriatric assessment rather than providing a definitive frailty diagnosis [8,9,10,11]. Vulnerability is a broader concept that encompasses biological, functional, psychological and social risk factors for poor outcomes; here it was assessed with the Vulnerable Elders Survey-13 (VES-13), which evaluates multidimensional geriatric vulnerability beyond physical frailty alone [12,13]. The conceptual relationships among these constructs, formulated a priori based on the study hypothesis, are illustrated in Appendix A, Figure A1.

Frailty screening scores and self-reported cognition scores frequently correlate, and it is tempting to read such a correlation as evidence that the two travel together. Depressive symptoms are common among older patients with cancer, lower scores on patient-reported cognitive measures, and are themselves associated with positive frailty screening, raising the possibility that part of the apparent frailty–cognition link is mediated by mood [14,15]. This distinction matters clinically because depressive symptoms, unlike frailty as a summary state, represent a potentially modifiable target.

This study had two objectives: first, to determine whether depressive symptoms, rather than G8 frailty screening status, were the strongest independent correlate of patient-reported cognitive function in older women with locally advanced breast cancer undergoing routine geriatric assessment; and second, as an exploratory analysis, to examine whether the overall geriatric profile remained consistent across successive periods of clinical practice spanning the pre-pandemic, pandemic/early post-pandemic, and contemporary guideline eras.

## 2. Materials and Methods

### 2.1. Study Design and Participants

This retrospective, cross-sectional analysis included all consecutive women aged ≥65 years with histopathologically confirmed locally advanced breast cancer (stage IIB–IIIC according to the AJCC 8th edition) who underwent a baseline geriatric assessment before initiation of oncological treatment. Only patients with complete data for all variables included in the primary analysis were retained for the final analysis. Patients with incomplete baseline geriatric or patient-reported outcome questionnaires, or with demographic or clinical data that were inconsistent and could not be reliably verified, were excluded from the complete-case analysis.

Although comprehensive geriatric assessment was routinely available for older patients regardless of disease stage, the present study was intentionally restricted to women with locally advanced disease to reduce clinical heterogeneity arising from differences in disease burden and treatment pathways, thereby allowing a more focused evaluation of the relationships among frailty screening status, depressive symptoms, and patient-reported cognitive function.

For exploratory temporal analyses, the study period was divided into three chronological cohorts: January 2018–December 2020, January 2021–December 2022, and January 2023–December 2025. These cohorts were not defined by changes in treatment protocols but were created to examine temporal consistency in geriatric assessment profiles across successive periods of routine clinical practice. The three cohorts were not intended to compare therapeutic strategies directly, and because this comparison was exploratory, no causal inference regarding temporal changes should be drawn.

The study was conducted in accordance with the Declaration of Helsinki and was approved by the Bagcilar Training and Research Hospital Non-Interventional Clinical Research Ethics Committee (Approval No. 2025/05/10/029, approved 10 December 2025). Because the analysis was retrospective, ethics committee approval was obtained before data extraction and statistical analysis, and only fully anonymized records were used.

### 2.2. Geriatric Assessment and Patient-Reported Outcomes

All assessments were performed at the initial geriatric oncology consultation, before any systemic or locoregional treatment had begun. No follow-up or post-treatment assessments were included; the dataset therefore represents a single cross-sectional evaluation per patient.

Frailty was screened with the G8 questionnaire, with a score of 14 or below taken as a positive screen indicating the need for further comprehensive geriatric assessment [8,9]. Geriatric vulnerability was assessed with the VES-13, which evaluates self-rated health, functional limitations and physical disabilities [12,13]. VES-13 was selected because it captures multidimensional geriatric vulnerability beyond physical frailty alone. Depressive symptoms were screened with the 15-item Geriatric Depression Scale (GDS), with a score of 5 or above taken as a positive screen [16,17]. Functional status was measured with the Lawton Instrumental Activities of Daily Living (IADL) scale [18,19].

Patient-reported cognitive function was assessed with the Functional Assessment of Cancer Therapy-Cognitive Function (FACT-Cog), which captures perceived cognitive difficulties as reported by the patient rather than objective neuropsychological performance [20,21]. General cancer-related quality of life was measured with the FACT-G [22,23], and global health status with the EORTC QLQ-C30 [24,25].

### 2.3. Data Handling

**Figure 1:**
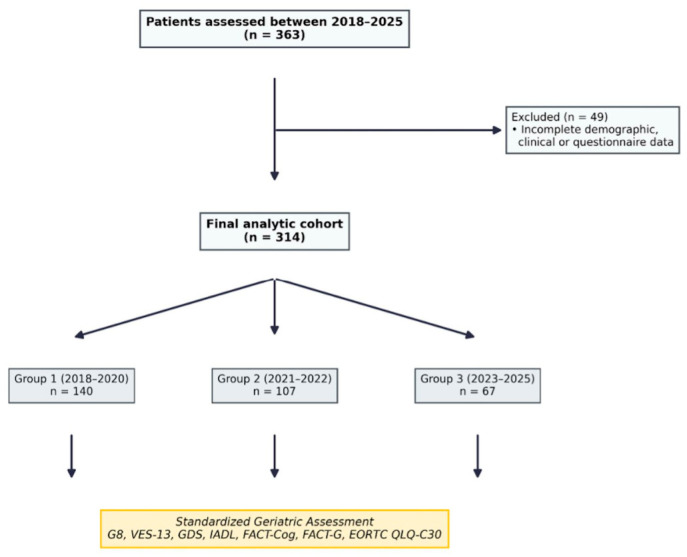
Study flow diagram. All consecutive older women with histologically confirmed locally advanced breast cancer who underwent routine geriatric assessment between 2018 and 2025 were screened for eligibility. Forty-nine records with incomplete demographic, clinical, or questionnaire data were excluded, leaving 314 patients for the primary analysis. For exploratory analyses, the final cohort was divided into three chronological treatment-era groups (2018–2020, 2021–2022, and 2023–2025).

Before any analysis, the database was screened for incomplete or inconsistent records. Of the 363 eligible patients, 49 were excluded because one or more baseline questionnaire assessments were incomplete or because key demographic/clinical data were inconsistent or could not be reliably verified, leaving 314 patients for the final analysis. The variables affected by missingness or inconsistency and their frequency are summarized in Appendix A, Table A1. Most exclusions resulted from incomplete geriatric or patient-reported outcome questionnaires, as some patients were unable or unwilling to complete all instruments. Because validated total scores cannot be calculated from incomplete questionnaires, and inconsistent demographic or staging data could not be reliably corrected, these records were not suitable for complete-case analysis. This process is also summarized in the study flow diagram (Figure 1).

### 2.4. Statistical Analysis

Continuous variables are summarized as mean ± standard deviation to facilitate comparison with previous geriatric oncology studies; because all continuous variables departed from normality on the Shapiro–Wilk test, non-parametric tests were used for all comparisons, and median-based summaries were inspected and showed the same between-group patterns. Distributions were assessed with the Shapiro–Wilk test; as all continuous variables departed significantly from normality, non-parametric tests were used throughout. Bivariate associations were assessed with Spearman’s rank correlation, with Pearson’s correlation reported alongside for the principal frailty-screening–cognition relationship. Two-group comparisons used the Mann–Whitney U test and three-group comparisons the Kruskal–Wallis test. Categorical comparisons used the chi-square test.

Because these geriatric instruments measure related aspects of the same underlying health domains, entering them into a single regression model introduced substantial multicollinearity. Accordingly, we fitted separate sensitivity models, each including age, GDS, and only one additional geriatric instrument (either VES-13 or IADL). In these models, endocrine therapy and radiotherapy represented the planned treatment strategy determined at the baseline multidisciplinary team conference before any systemic or locoregional treatment had begun, rather than treatments received after the FACT-Cog assessment. Detailed results of the sensitivity models are presented in Appendix A, Table A2.

Regression assumptions were formally evaluated. Residual normality was assessed by the Shapiro–Wilk test and visual inspection of Q–Q plots. Homoscedasticity was tested with the Breusch–Pagan test. Multicollinearity was evaluated through variance inflation factors (VIF). Independence of residuals was assessed with the Durbin–Watson statistic. Influential observations were examined with Cook’s distance. Standardized coefficients (β), standard errors, 95% confidence intervals and exact *p*-values are reported. A two-sided *p* < 0.05 was considered significant. Analyses were performed in IBM SPSS Statistics (version 29).

## 3. Results

### 3.1. Cohort Characteristics

The analytic cohort comprised 314 women with a mean age of 72.9 ± 5.6 years. Tumors were predominantly luminal (Luminal A 67.8%, Luminal B 24.5%, HER2-enriched 7.6%); no triple-negative tumors were present. All patients had locally advanced disease (stage IIB–IIIC). The three exploratory treatment-era cohorts contained 140, 107 and 67 patients respectively. Full demographic and clinical characteristics by cohort, including between-cohort *p*-values, are shown in Table 1.

### 3.2. Prevalence of Positive Frailty Screening and Depressive Symptoms

A positive frailty screen (G8 ≤ 14) was found in 295 of 314 patients (93.9%); only 19 patients (6.1%) had a negative screen. One hundred and two patients (32.5%) screened positive for depressive symptoms (GDS ≥ 5). Lower patient-reported cognitive function, defined descriptively as a FACT-Cog score falling in the lowest quartile of the cohort (≤100), was observed in 44 patients (14.0%). We selected the lowest quartile only for descriptive purposes; no validated FACT-Cog cut-off exists for classifying cognitive difficulty in older breast cancer patients. Given this limitation, FACT-Cog was analyzed as a continuous outcome throughout the main analysis.

### 3.3. Relationship Between Frailty Screening Status and Patient-Reported Cognitive Function

**Figure 2:**
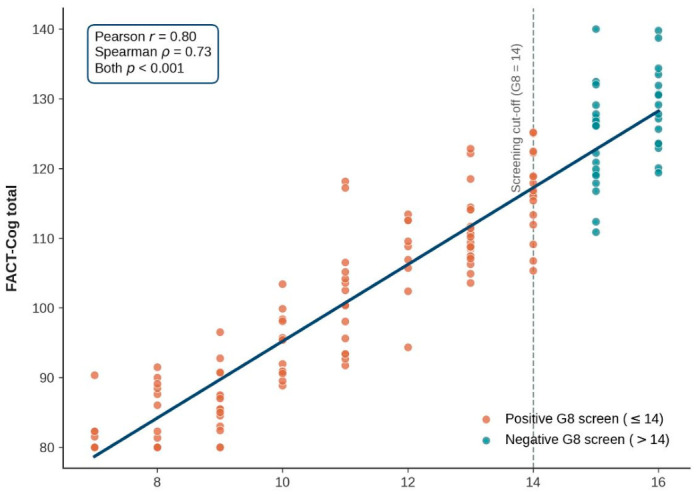
Scatter Plot Association between G8 screening score and patient-reported cognitive function (FACT-Cog). Scatter plot demonstrating the relationship between continuous G8 screening score and FACT-Cog total score. The solid line represents the linear regression fit. The vertical dashed line indicates the predefined G8 screening cut-off (14), separating patients with positive (≤14) and negative (>14) G8 screening results. Pearson and Spearman correlation coefficients are shown.

G8 screening score was strongly correlated with FACT-Cog (Pearson r = 0.80, Spearman ρ = 0.73; both *p* < 0.001; Figure 2).

**Figure 3:**
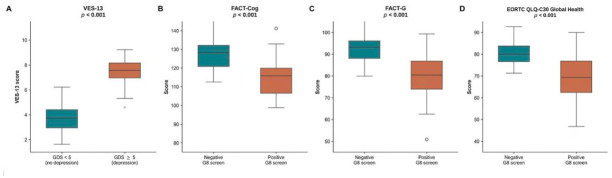
Boxplots according to depressive symptom status and G8 screening status. Boxplots illustrating differences in geriatric vulnerability and patient-reported outcomes according to depressive symptom status and G8 screening status. (**A**) VES-13 scores according to depressive symptom status (GDS < 5 vs. GDS ≥ 5). (**B**) FACT-Cog scores according to G8 screening status (negative > 14 vs. positive ≤ 14). (**C**) FACT-G scores according to G8 screening status. (**D**) EORTC QLQ-C30 Global Health scores according to G8 screening status. Horizontal lines indicate medians, boxes represent the interquartile range (IQR), and whiskers indicate the data range excluding outliers.

Patients with a positive frailty screen reported substantially lower cognitive function than those with a negative screen (FACT-Cog 113.0 vs. 129.0, Mann–Whitney U, *p* < 0.001). They also reported significantly lower FACT-G and EORTC QLQ-C30 global health scores (Figure 3B–D). These comparisons should, however, be interpreted cautiously given the small size of the negative-screen group (n = 19, 6.1%).

### 3.4. Association of Depressive Symptoms with Patient-Reported Cognitive Function

The bivariate picture changed substantially once depressive symptoms were taken into account. In the multivariable regression model (FACT-Cog regressed on G8, age and GDS), depressive symptom burden was by far the strongest independent correlate of patient-reported cognitive function (B = −5.14, β = −0.89, *p* < 0.001). The overall model explained 89.7% of the variance in FACT-Cog (adjusted R^2^ = 0.896, F = 902.9, *p* < 0.001), whereas G8 screening score was no longer independently associated with patient-reported cognition (B = 0.32, β = 0.05, *p* = 0.16). Age showed no statistically significant independent association (B = −0.08, *p* = 0.07).

Regression diagnostics were satisfactory: VIF ranged from 1.16 to 3.56 (no multicollinearity); the Durbin–Watson statistic was 1.78 (no autocorrelation); the Breusch–Pagan test was non-significant (LM = 1.96, *p* = 0.58), confirming homoscedasticity; no influential observations were identified (maximum Cook’s distance = 0.06). Residual normality was rejected by the Shapiro–Wilk test (W = 0.93, *p* < 0.001), but visual inspection of the Q–Q plot showed only mild deviation in the tails, and with N = 314, OLS estimates remain robust to this degree of non-normality. Full regression results, including unstandardized coefficients, standardized coefficients, standard errors, t statistics, 95% confidence intervals, and model statistics, are presented in Table 2.

In separate sensitivity models that combined age and GDS with one alternative geriatric instrument, depressive symptoms remained a strong and highly significant independent correlate of FACT-Cog (GDS standardized β between −0.44 and −0.57, all *p* < 0.001; Appendix A, Table A2). In the model containing VES-13, depressive symptoms and VES-13 were of similar magnitude (β = −0.44 and −0.52, respectively), whereas in the model containing IADL and in the primary model, GDS was the single strongest correlate. For completeness, we also fitted a combined model containing all overlapping instruments simultaneously; in that model, the variance inflation factors reached 20.0, indicating that the individual coefficients were unstable, so this model is reported for completeness only and is not interpreted as confirmatory. Across every model, G8 screening score remained non-significant (*p* ≥ 0.16), consistent with the primary analysis.

Consistent with the regression findings, patients with depressive symptoms (GDS ≥ 5, n = 102) had significantly higher VES-13 vulnerability scores than those without depressive symptoms (7.8 vs. 3.6, Mann–Whitney U, *p* < 0.001; Figure 3A).

### 3.5. Exploratory Comparison Across Treatment Eras

The predefined treatment-era comparison was exploratory and intended to evaluate temporal stability rather than to compare therapeutic strategies. Frailty screening scores, depressive symptom burden, vulnerability, patient-reported cognitive function, general quality of life and global health status did not differ significantly across the three cohorts (all Kruskal–Wallis *p* > 0.05); IADL showed a statistically significant but modest difference (*p* = 0.014). As this comparison was exploratory, no causal inference regarding temporal changes should be made. Detailed results are summarized in Appendix A, Table A3.

## 4. Discussion

In this consecutive series of older women with locally advanced breast cancer assessed at two tertiary centers between 2018 and 2025, a positive frailty screen was almost universal and tracked closely with lower patient-reported cognitive function. The principal finding emerged after multivariable adjustment: once depressive symptoms were accounted for, the independent contribution of G8 screening score to patient-reported cognition was no longer statistically significant. In this dataset, the apparent relationship between frailty screening status and patient-reported cognitive function was largely attenuated after adjustment for depressive symptoms.

Although biologically plausible, this finding should be interpreted cautiously. Both the GDS and the FACT-Cog are patient-reported instruments, and the strong association between them may partly reflect common method variance—that is, shared subjective-response tendencies, affective coloring of self-appraisal, and overlapping item content (depressive symptoms include concentration difficulty, which is also captured by FACT-Cog). The high explained variance (R^2^ = 0.90) should therefore be interpreted cautiously; it likely reflects construct overlap between self-report instruments as well as a genuine clinical relationship. The model should not be interpreted as explaining objective cognitive performance but rather variation in patient-reported cognitive function. We do not claim that depression is more important than frailty for objective cognition, which was not measured in this study.

Previous studies have consistently reported associations between depressive symptoms and subjective cognitive complaints in both older adults and patients with cancer [26,27]. These associations are generally stronger for patient-reported cognitive measures than for objective neuropsychological performance, supporting the concept that mood substantially influences perceived cognitive function [28]. Our findings extend these observations by demonstrating that, within a cohort of older women with locally advanced breast cancer, depressive symptoms remained the strongest independent correlate of patient-reported cognitive function even after adjustment for age and G8 frailty screening score.

The prevalence of lower patient-reported cognitive function in our cohort (14.0% in the lowest FACT-Cog quartile) was lower than the 25–50% reported for subjective cognitive complaints in general older populations [21] and the 20–40% described in breast cancer survivors exposed to chemotherapy [22]. This likely reflects the more detailed multi-domain nature of the FACT-Cog questionnaire and the pre-treatment assessment timing. It is also possible that some older patients may underestimate their cognitive difficulties on a self-report measure, a phenomenon well described in the geriatric literature [24].

Despite these limitations, the clinical implication is nonetheless worth noting. A positive frailty screen tells the clinician that a patient is vulnerable but does not, on its own, point to a specific intervention. A positive depression screen, by contrast, identifies a potentially modifiable condition. If lower self-reported cognition in these women is partly a marker of untreated depressive symptoms, a brief mood screen should be considered an integral component of routine geriatric assessment and may represent a clinically actionable target for prospective evaluation.

The modest difference observed in IADL scores may reflect differences in patient case-mix across the three periods rather than clinically meaningful changes in functional status. Importantly, this isolated finding was not accompanied by differences in the remaining geriatric assessment domains. The three cohorts were not intended to compare treatment strategies directly but rather to evaluate whether the geriatric profile of older patients remained generally comparable despite substantial changes in clinical practice, including temporary service disruptions during the COVID-19 pandemic and subsequent implementation of contemporary geriatric oncology recommendations [2,3]. The comparison across treatment eras was exploratory and intended to describe overall geriatric assessment profiles rather than to formally evaluate whether the relationship between depressive symptoms and patient-reported cognitive function changed over time.

Several limitations should be acknowledged. First, this is a cross-sectional study, so we cannot determine the direction of the relationship between depressive symptoms and patient-reported cognitive function; prospective and intervention studies are needed. Second, cognition was assessed by patient self-report (FACT-Cog) rather than by objective neuropsychological testing, and self-reported cognition is itself sensitive to mood; the high R^2^ partly reflects this construct overlap. Third, the G8 is a frailty screening tool, not a definitive frailty classification; our findings relate to frailty screening status rather than to a comprehensive frailty phenotype or frailty index. Fourth, the fit group comprised only 19 patients (6.1%), so comparisons between patients with positive and negative frailty screens should be interpreted cautiously due to possible instability. Fifth, education level, comorbidity burden, anxiety symptoms, antidepressant use and sleep quality were not collected and could not be included as covariates. Residual confounding by unmeasured variables cannot be excluded. Sixth, the treatment-era cohorts were unequal in size (140, 107 and 67 patients) because they reflected the number of consecutively assessed patients available during each predefined institutional period. Finally, because the study was restricted to women with locally advanced breast cancer treated at two tertiary referral centers, the findings may not be fully generalizable to patients with early-stage disease or other healthcare settings.

## 5. Conclusions

In this cross-sectional analysis of older women with locally advanced breast cancer, depressive symptoms were the strongest independent correlate of patient-reported cognitive function, whereas G8 frailty screening score was no longer independently associated after multivariable adjustment. These findings support the routine integration of brief depression screening into geriatric assessment while highlighting the need for prospective studies incorporating objective neuropsychological testing to clarify causal relationships.

## Figures and Tables

**Table 1:** Demographic and Clinical Characteristics by Cohort.

Characteristic	Cohort 1 (2018–2020) n = 140	Cohort 2 (2021–2022) n = 107	Cohort 3 (2023–2025) n = 67	Overall n = 314
Age, years (mean ± SD)	72.0 ± 4.8	72.9 ± 4.7	74.9 ± 7.7	72.9 ± 5.6
**Molecular subtype, n (%)**				
Luminal A	84 (60.0%)	84 (78.5%)	45 (67.2%)	213 (67.8%)
Luminal B	46 (32.9%)	16 (15.0%)	15 (22.4%)	77 (24.5%)
Her2-Enriched	10 (7.1%)	7 (6.5%)	7 (10.4%)	24 (7.6%)
**AJCC Stage, n (%)**				
IIB	53 (37.9%)	51 (47.7%)	22 (32.8%)	126 (40.1%)
IIIA	51 (36.4%)	35 (32.7%)	29 (43.3%)	115 (36.6%)
IIIB	11 (7.9%)	10 (9.3%)	11 (16.4%)	32 (10.2%)
IIIC	25 (17.9%)	11 (10.3%)	5 (7.5%)	41 (13.1%)
**Histologic Grade, n (%)**				
G1	75 (53.6%)	44 (41.1%)	38 (56.7%)	157 (50.0%)
G2	34 (24.3%)	48 (44.9%)	22 (32.8%)	104 (33.1%)
G3	31 (22.1%)	15 (14.0%)	7 (10.4%)	53 (16.9%)
**RT, n (%)**				
Received RT	88 (62.9%)	67 (62.6%)	39 (58.2%)	194 (61.8%)
**ET, n (%)**				
Received ET	112 (80.0%)	95 (88.8%)	47 (70.1%)	254 (80.9%)
**Adjuvant regimen, n (%)**				
AC	21 (15.0%)	24 (22.4%)	6 (9.0%)	51 (16.2%)
AC + Paclitaxel	62 (44.3%)	46 (43.0%)	29 (43.3%)	137 (43.6%)
Paclitaxel	1 (0.7%)	2 (1.9%)	0 (0.0%)	3 (1.0%)
Transtuzumab	2 (1.4%)	1 (0.9%)	0 (0.0%)	3 (1.0%)
Docetaxel	12 (8.6%)	10 (9.3%)	2 (3.0%)	24 (7.6%)
FEC	13 (9.3%)	8 (7.5%)	6 (9.0%)	27 (8.6%)
AC + Transtuzumab	10 (7.1%)	9 (8.4%)	18 (26.9%)	37 (11.8%)
None	19 (13.6%)	7 (6.5%)	6 (9.0%)	32 (10.2%)

Values are presented as n (%) unless otherwise indicated. Between-cohort comparisons were performed using the chi-square test for categorical variables and the Kruskal–Wallis test for continuous variables. No triple-negative tumors were present in the study cohort. AC: Adriamycin + Cyclophosphamide; AJCC: American Joint Committee on Cancer; ET: Endocrine Treatment; FEC: Fluorouracil + Epirubicin + Cyclophosphamide; RT: Radiotherapy.

**Table 2:** Multivariable linear regression model for patient-reported cognitive function (FACT-Cog).

Predictor	B (95% CI)	SE	Standardized β	t	*p*-Value
**GDS score**	−5.14 (−5.53 to −4.75)	0.198	−0.89	−26.02	<0.001
**G8 screening score**	0.32 (−0.12 to 0.75)	0.222	0.05	1.42	0.156
**Age (years)**	−0.08 (−0.17 to 0.01)	0.045	−0.04	−1.83	0.068
**Model Statistic**				**Value**	
Adjusted R^2^				0.896	
F statistic				902.9	
Model *p*				<0.001	
Durbin–Watson				1.78	

Dependent variable: FACT-Cog total score. The multivariable linear regression model included GDS score, G8 screening score, and age as prespecified covariates. Standardized regression coefficients (β) are reported to facilitate comparison of the relative contribution of each predictor. B: Unstandardized Regression Coefficient; CI: Confidence Interval; SE: Standard Error; β: Standardized Regression Coefficient. Predictor-specific variance inflation factors were all below the conventional threshold of 5 (GDS = 3.56, G8 = 3.32, age = 1.16; range 1.16–3.56), confirming the absence of problematic multicollinearity.

## Data Availability

The datasets generated and/or analyzed during the current study are not publicly available to protect patient confidentiality but are available from the corresponding author on reasonable request.

## Abbreviations

The following abbreviations are used in this manuscript:

AC	Adriamycin + Cyclophosphamide
AJCC	American Joint Committee on Cancer
**β**	Standardized Regression Coefficient
B	Unstandardized Regression Coefficient
CI	Confidence Interval
EORTC QLQ-C30	European Organization for Research and Treatment of Cancer Quality of Life Questionnaire Core 30
ET	Endocrine Treatment
FACT-Cog	Functional Assessment of Cancer Therapy—Cognitive Function
FACT-G	Functional Assessment of Cancer Therapy—General
FEC	Fluorouracil + Epirubicin + Cyclophosphamide
GDS	Geriatric Depression Scale
IADL	Instrumental Activities of Daily Living
KW	Kruskal–Wallis
NS	No Statistically Significant Difference
RT	Radiotherapy
SD	Standard Deviation
SE	Standard Error
VES-13	Vulnerable Elders Survey-13
VIF	Variance Inflation Factor

## Appendix A

**Table A1:** Reasons for exclusion due to missing data.

**Variable Affected**	**n**
**FACT-Cog**	21
**GDS**	14
**G8**	8
**VES-13**	18
**FACT-G**	10
**EORTC QLQ-C30**	12
**Inconsistent clinical staging data**	2
**Inconsistent demographic data**	1

Some patients had more than one missing or inconsistent variable; therefore, the total number of affected variables exceeds the total number of excluded patients (n = 49).

**Table A2:** Sensitivity multivariable linear regression models for patient-reported cognitive function (FACT-Cog).

**Model 2a—**FACT-Cog ~ age + GDS + VES-13 Model Fit: R^2^ = 0.920; adjusted R^2^ = 0.919; F(3,310) = 1190.9, *p* < 0.001						
**Predictor**	**B**	**SE**	**β**	**95% CI**	***p***	**VIF**
GDS score	−2.53	0.314	−0.44	(−3.15, −1.91)	<0.001	11.5
VES-13 score	−2.71	0.283	−0.52	(−3.27, −2.15)	<0.001	11.4
Age, years	−0.08	0.039	−0.03	(−0.15, 0.00)	0.056	1.16
**Model 2b—**FACT-Cog ~ age + GDS + IADL Model Fit: R^2^ = 0.927; adjusted R^2^ = 0.927; F(3,310) = 1319.1, *p* < 0.001						
**Predictor**	**B**	**SE**	**β**	**95% CI**	***p***	**VIF**
GDS score	−3.26	0.208	−0.57	(−3.67, −2.85)	<0.001	5.6
IADL score	4.64	0.405	0.41	(3.84, 5.44)	<0.001	5.6
Age, years	−0.04	0.038	−0.02	(−0.11, 0.04)	0.344	1.17
**Model 2c—**Combined Model (all overlapping instruments; reported for completeness only) Model Fit: R^2^ = 0.932; adjusted R^2^ = 0.930; F(8,305) = 518.9, *p* < 0.001						
**Predictor**	**B**	**SE**	**β**	**95% CI**	***p***	**VIF**
GDS score	−2.70	0.319	−0.47	(−3.33, −2.08)	<0.001	13.7
G8 screening score	−0.24	0.194	−0.04	(−0.63, 0.14)	0.209	3.7
VES-13 score	−1.15	0.350	−0.22	(−1.84, −0.47)	0.001	20.0
IADL score	3.58	0.544	0.32	(2.51, 4.65)	<0.001	10.5
Age, years	−0.04	0.039	−0.02	(−0.12, 0.03)	0.260	1.3
Disease stage	0.28	0.198	0.02	(−0.11, 0.67)	0.163	1.1
Planned endocrine therapy	0.84	0.505	0.03	(−0.16, 1.83)	0.098	1.1
Planned radiotherapy	−0.04	0.406	0.00	(−0.84, 0.76)	0.916	1.1

Dependent variable: FACT-Cog. Models 2a and 2b are the preferred, reduced-collinearity sensitivity analyses (each geriatric instrument tested separately). Model 2c contains all overlapping instruments simultaneously and is shown only for completeness; its VIF values reach 20.0, so its individual coefficients are unstable and are not interpreted as confirmatory. Endocrine therapy and radiotherapy denote the planned treatment strategy set at the baseline multidisciplinary team conference. B: Unstandardized Coefficient; SE: Standard Error; β: Standardized Coefficient; CI: Confidence Interval; VIF: Variance Inflation Factor.

**Table A3:** Geriatric assessment and patient-reported outcome measures according to treatment-era cohort.

	Cohort 1 (2018–2020) n = 140	Cohort 2 (2021–2022) n = 107	Cohort 3 (2023–2025) n = 67	Overall n = 314	*p*-Value KW
**Age (Years)**	72.0 ± 4.8	72.9 ± 4.7	74.9 ± 7.7	72.9 ± 5.6	0.05
**G8 score**	12.5 ± 1.7	12.3 ± 1.5	12.3 ± 2.8	12.4 ± 1.9	0.19
**GDS (Baseline)**	3.5 ± 2.0	3.9 ± 2.1	3.9 ± 2.8	3.7 ± 2.2	0.497
**VES-13**	4.9 ± 2.2	4.7 ± 2.2	5.6 ± 3.1	5.0 ± 2.5	0.194
**IADL**	6.8 ± 1.0	7.1 ± 0.9	6.4 ± 1.5	6.8 ± 1.1	0.014
**FACT-Cog**	115.0 ± 11.9	114.3 ± 10.3	111.4 ± 17.4	114.0 ± 12.8	0.615
**FACT-G**	81.3 ± 9.9	80.6 ± 9.0	77.8 ± 14.2	80.3 ± 10.7	0.405
**QLQ-C30 Global**	69.8 ± 10.2	69.3 ± 9.7	65.5 ± 14.5	68.7 ± 11.2	0.220

Values are presented as mean ± standard deviation. Between-cohort comparisons were performed using the Kruskal–Wallis test. Only IADL differed significantly across cohorts (*p* = 0.014); all other geriatric assessment and patient-reported outcome measures showed no statistically significant between-cohort difference. KW: Kruskal–Wallis.
